# Supplementary material for: Ethylene-Propylene-Methylene/Isoprene Rubber/SiO2 Nanocomposites with Enhanced Mechanical Performances and Deformation Recovery Ability by a Combination of Synchronously Vulcanizing and Nanoparticle Reinforcement
Source: Polymers (Basel). 2024 Oct 3;16(19):2809. doi: 10.3390/polym16192809 (PMC11478894; doi:10.3390/polym16192809)
Supplement: Supplementary file 1 [file polymers-16-02809-s001.zip › polymers-3228390-supplementary.pdf]

Supplementary Information for

Ethylene-Propylene-Methylene/Isoprene Rubber/SiO_2 Nanocomposites with Enhanced Mechanical Performances and Deformation Recovery Ability by a Combination of Synchronously Vulcanizing and Nanoparticle Reinforcement

Rongyan Hu [1], Ran Xiao [1], Xinxin Xia [1], Yonggang Shangguan [1,2,*] and Qiang Zheng [1,2]

1 MOE Key Laboratory of Macromolecule Synthesis and Functionalization, Department of Polymer Science and Engineering, Zhejiang University, Hangzhou 310027, China; 12229007@zju.edu.cn (R.H.); 2023310064@link.tyut.edu.cn (R.X.); 21629048@zju.edu.cn (X.X.); zhengqiang@zju.edu.cn (Q.Z.)
2 Shanxi-Zheda Institute of Advanced Materials and Chemical Engineering, Taiyuan 030032, China
* Correspondence: shangguan@zju.edu.cn; Tel.: +86-0571-8795-3075; Fax: +86-0571-8795-3075

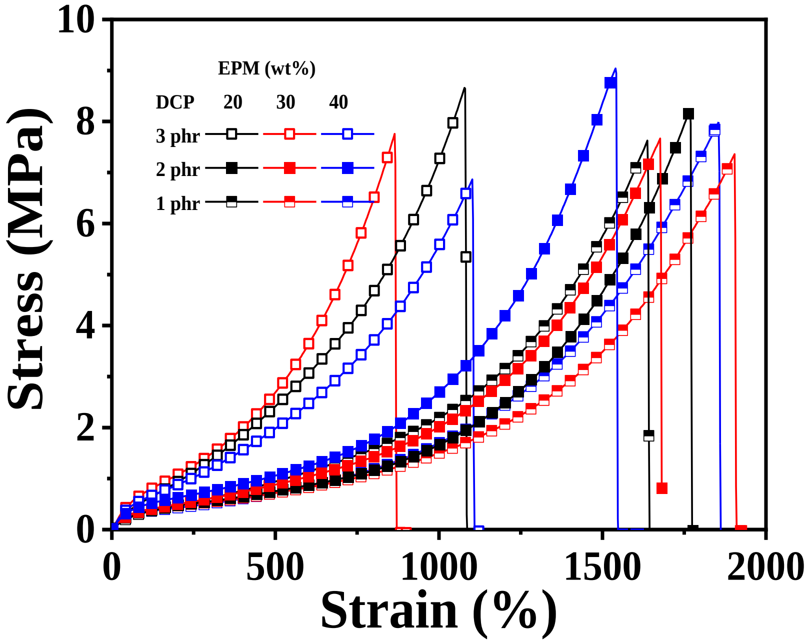

Figure S1. Tensile curves of EPM/IR compound vulcanizates with various content of DCP.

Table S1. Tensile parameters of EPM/IR vulcanizates with various compositions.

Weight Fraction of EPM (%)	Breaking Strength (MPa)	Breaking Strain (%)	Modulus (MPa)	Toughness (MJ/m³)
0	3.3 ± 0.8	652.0 ± 140	0.67 ± 0.18	10.16 ± 4.07
20	8.2 ± 1.9	1767 ± 101	0.79 ± 0.02	55.32 ± 13.43
30	7.7 ± 0.8	1495 ± 89	0.96 ± 0.03	47.72 ± 4.91
40	9.0 ± 0.9	1540 ± 83	0.92 ± 0.01	50.11 ± 7.87
50	5.5 ± 1.1	1453 ± 191	1.03 ± 0.02	33.51 ± 11.16
60	3.0 ± 0.1	1252 ± 22	1.18 ± 0.05	17.61 ± 0.70
70	2.8 ± 0.3	1184 ± 41	1.34 ± 0.03	16.57 ± 1.65
80	0.7 ± 0.1	1038 ± 141	1.24 ± 0.03	6.29 ± 1.26

100	1.7 ± 0.1	1738 ± 48	1.53 ± 0.02	16.53 ± 0.39

Table S2. Tensile parameters of EPM40 with different Silica content.

Silica Content (phr)	Breaking Strength (MPa)	Breaking Strain (%)	Modulus (MPa)	Toughness (MJ/m³)
10	6.5 ± 0.9	1678 ± 83	0.92 ± 0.01	50.11 ± 7.87
20	10.7 ± 3.2	1273 ± 131	1.42 ± 0.003	46.33 ± 15.27
30	12.4 ± 0.5	1109 ± 7	2.00 ± 0.05	55.16 ± 3.00
40	3.5 ± 0.5	203 ± 45	2.58 ± 0.20	3.85 ± 0.97

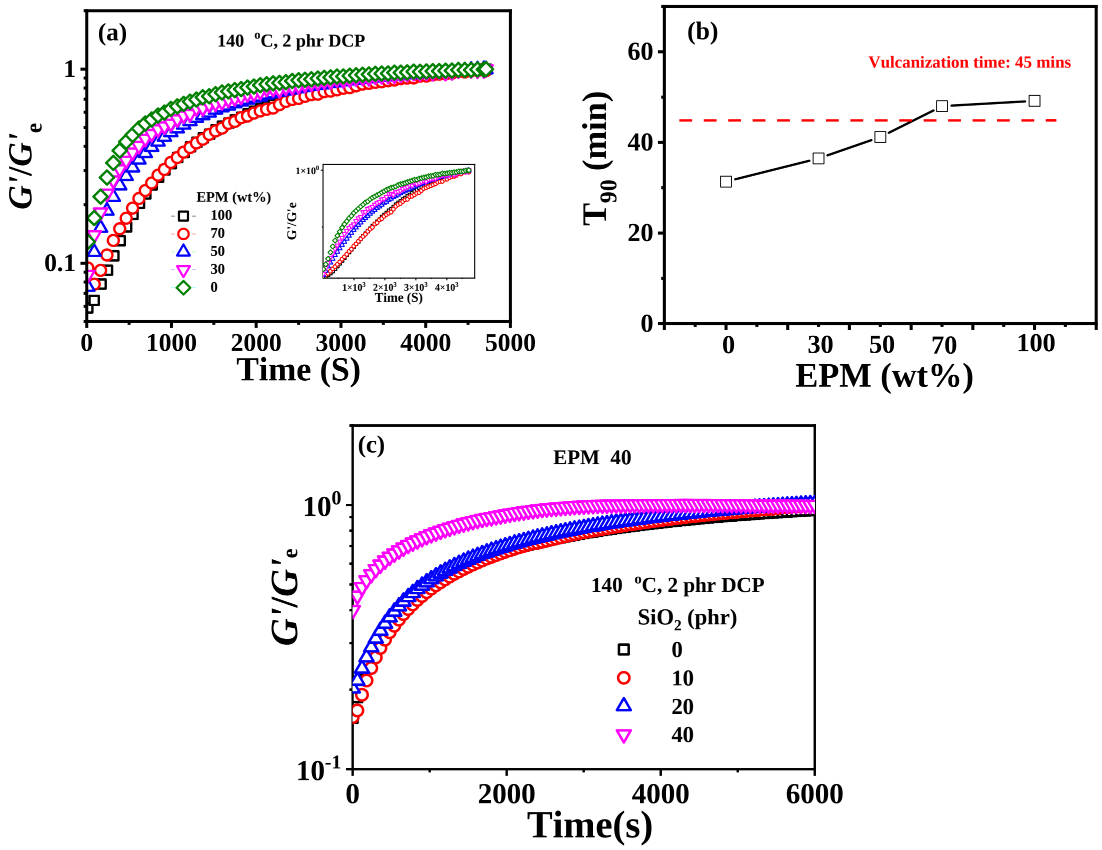

Figure S2. (**a**) Curing curves with linear coordinates inside and (**b**) Optimized curing time T_{90} of various EPM/IR vulcanizates with 2 phr DCP at 140 °C. (**c**) Curing curves of EPM/IR/silica vulcanizates with different silica loading.

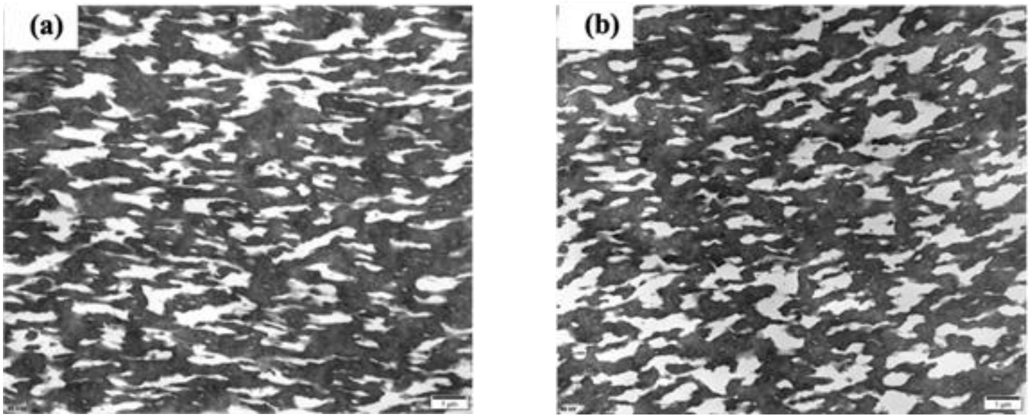

Figure S3. TEM micrographs of vulcanizated EPR/IR/Silica composites with different silica laoding: (**a**) 20 phr and (**b**) 40 phr. Composition of EPR/IR in composites is fixed at 40:60 in weight.

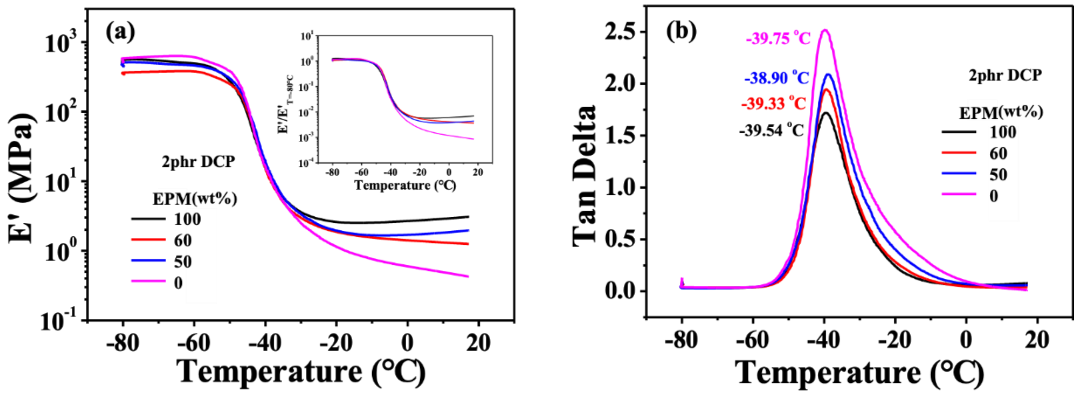

Figure S4. Temperature dependence of (**a**) dynamic modulus and (**b**) loss factor for various EPM/IR compound vulcanizates.

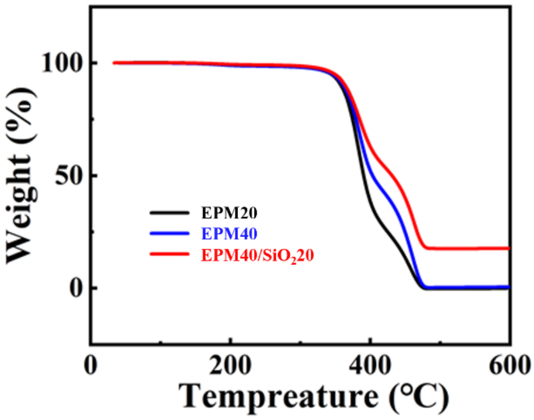

Figure S5. Thermogravimetric analysis curves of the different EPM/IR/silica vulcanizates.

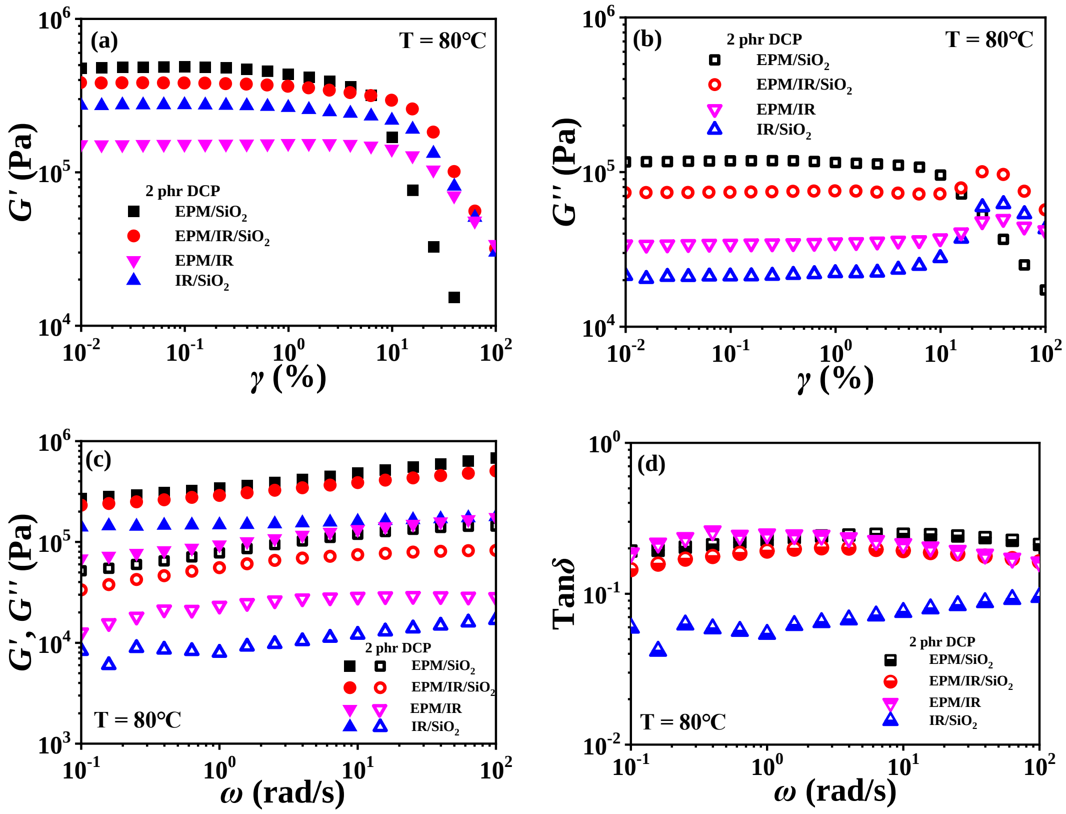

Figure S6. (**a**,**b**) Strain sweep curves and (**c**,**d**) frequency sweep curves of different EPM/IR/silica vulcanizates at 80 °C.

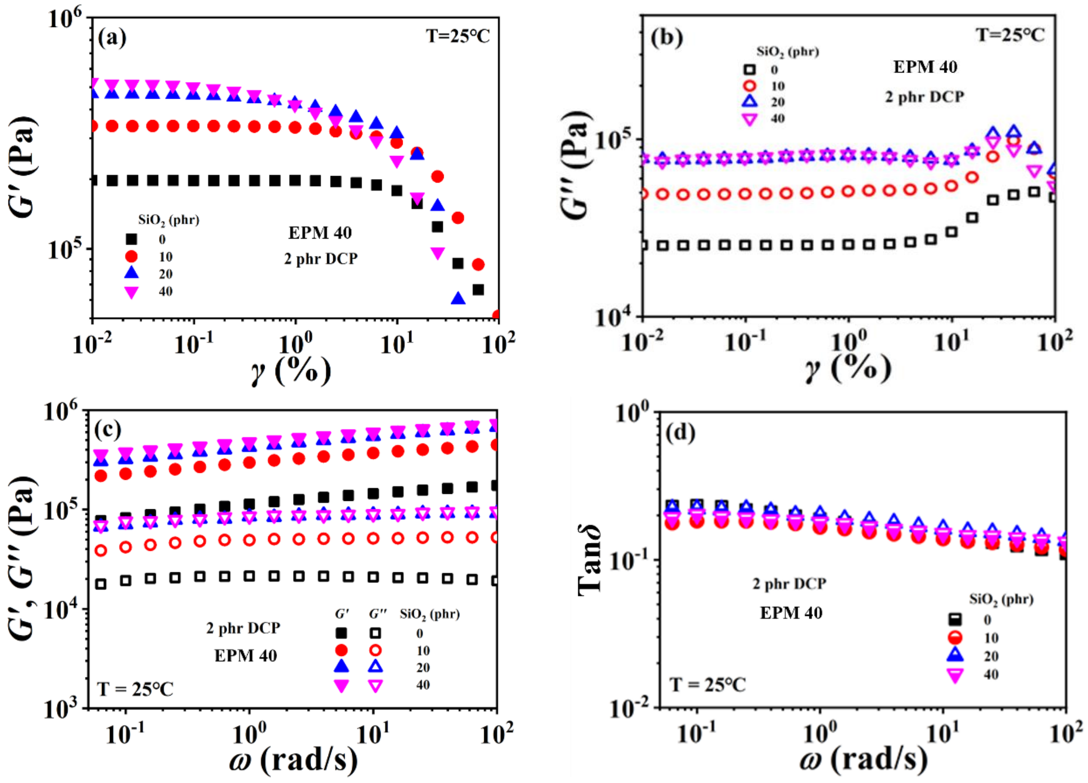

Figure S7. (**a**,**b**) Strain sweep curves and (**c**,**d**) frequency sweep curves of EPM/IR/silica vulcanizates with different silica loading at 25 °C.

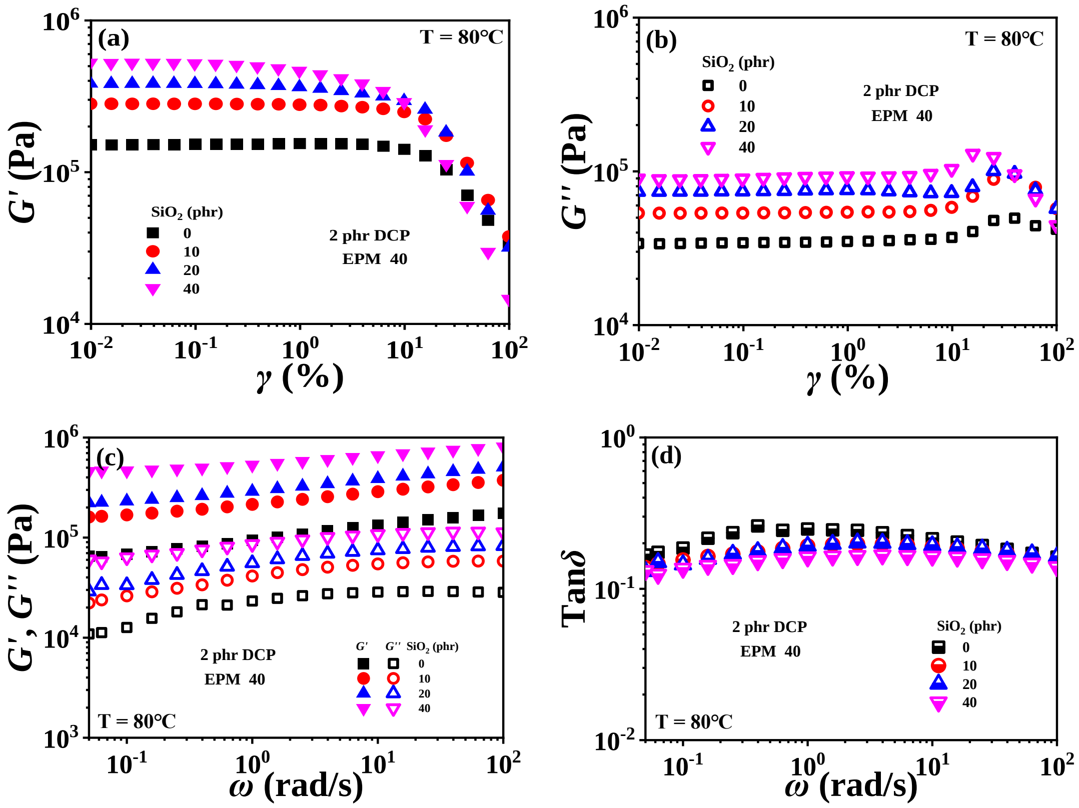

Figure S8. (**a**,**b**) Strain sweep curves and (**c**,**d**) frequency sweep curves of EPM/IR/silica vulcanizates with different silica loading at 80 °C.
